# Endotoxin leads to rapid subcellular re-localization of hepatic RXRα: A novel mechanism for reduced hepatic gene expression in inflammation

**DOI:** 10.1186/1478-1336-2-4

**Published:** 2004-08-16

**Authors:** Romi Ghose, Tracy L Zimmerman, Sundararajah Thevananther, Saul J Karpen

**Affiliations:** 1 Texas Children's Liver Center, Department of Pediatrics, Baylor College of Medicine, One Baylor Plaza, Houston, TX 77030, USA

**Keywords:** liver, inflammation, endotoxin, RXR, JNK, nuclear export, nuclear receptor

## Abstract

**Background:**

Lipopolysaccharide (LPS) treatment of animals down-regulates the expression of hepatic genes involved in a broad variety of physiological processes, collectively known as the negative hepatic acute phase response (APR). Retinoid X receptor α (RXRα), the most highly expressed RXR isoform in liver, plays a central role in regulating bile acid, cholesterol, fatty acid, steroid and xenobiotic metabolism and homeostasis. Many of the genes regulated by RXRα are repressed during the negative hepatic APR, although the underlying mechanism is not known. We hypothesized that inflammation-induced alteration of the subcellular location of RXRα was a common mechanism underlying the negative hepatic APR.

**Results:**

Nuclear RXRα protein levels were significantly reduced (~50%) within 1–2 hours after low-dose LPS treatment and remained so for at least 16 hours. RXRα was never detected in cytosolic extracts from saline-treated mice, yet was rapidly and profoundly detectable in the cytosol from 1 hour, to at least 4 hours, after LPS administration. These effects were specific, since the subcellular localization of the RXRα partner, the retinoic acid receptor (RARα), was unaffected by LPS. A potential cell-signaling modulator of RXRα activity, c-Jun-N-terminal kinase (JNK) was maximally activated at 1–2 hours, coincident with maximal levels of cytoplasmic RXRα. RNA levels of RXRα were unchanged, while expression of 6 sentinel hepatic genes regulated by RXRα were all markedly repressed after LPS treatment. This is likely due to reduced nuclear binding activities of regulatory RXRα-containing heterodimer pairs.

**Conclusion:**

The subcellular localization of native RXRα rapidly changes in response to LPS administration, correlating with induction of cell signaling pathways. This provides a novel and broad-ranging molecular mechanism for the suppression of RXRα-regulated genes in inflammation.

## Background

LPS, a major constituent of the outer membrane of gram-negative bacteria, potently stimulates host innate immune response [1]. LPS-induced activation of monocytes/macrophages leads to the release of proinflammatory cytokines such as interleukin-1β (IL-1β), interleukin-6 (IL-6), and tumor necrosis factor-α (TNFα) in addition to other mediators such as cysteinyl leukotrienes [2]. LPS and LPS-induced cytokines have been implicated in the pathogenesis and progression of a variety of liver diseases, including cholestasis, as well as being principal mediators of the negative hepatic APR [3]. The cholestatic effect of LPS is primarily due to cytokine-mediated inhibition of the function and expression of hepatic genes encoding critical proteins involved in bile formation and transport (reviewed in [4]). These hepatocellular transporters include the basolateral sodium/taurocholate cotransporter (*Ntcp/Slc10a1*) and organic anion transporting proteins (*Oatp1/Slc21a1*), as well as the canalicular multispecific organic anion exporter (*Mrp2/Abcc2*) and the bile salt export protein (*Bsep/Abcb11*). Transcriptional down-regulation of the principal hepatic bile acid importer, Ntcp contributes to the reduction in bile acid uptake by hepatocytes in inflammation, whereas reduced Mrp2 expression leads to impaired excretion of conjugated bilirubin, glutathione and other organic anions into bile [5,6].

Recent reports have provided insights into the link between inflammation-mediated cell signaling and regulation of bile acid homeostasis in the liver. Geier *et al*. showed that LPS-mediated suppression of *Ntcp *RNA was almost completely blocked by pre-treatment with anti-IL-1β specific antibodies, indicating that the cholestatic effects of LPS on the expression of this gene may be primarily mediated by the cell signaling pathways initiated by this one cytokine [7]. We have shown that IL-1β treatment of HepG2 cells, or primary rat hepatocytes, leads to JNK-dependent repression of nuclear binding activity of the *Ntcp *transactivator, RXRα:RARα, with consequent down-regulation of *Ntcp *promoter activity [8]. Finally, LPS, cytokines, and activated JNK have been linked to reduced expression of the rate-limiting enzyme in the bile acid biosynthetic pathway, cholesterol 7α-hydroxylase (CYP7A1), thus linking inflammatory signaling in the liver to the known and coordinated suppression of both bile acid import and synthesis [9-11]. How activated JNK leads to reduced RXRα function is not known, but is likely to involve direct phosphorylation of RXRα [8].

Phosphorylation of nuclear receptors (NRs) is a rapid and potentially powerful means of regulating NR activity, that, depending upon the NR, can affect transcriptional activity, protein stability, sub-cellular localization, protein-protein interactions or DNA binding activity [12,13]. Phosphorylation of transfected RXRα was reported to alter its transactivation properties in vitro, however, a definite functional role for native RXRα phosphorylation remains controversial. Both enhanced and reduced proteasome-mediated RXRα degradation have been associated with RXRα phosphorylation [14,15]. Hyperphosphorylation of RXRα by JNK was reported by Adam-Stitah *et al *[16] and the phosphorylation sites were mapped to several residues (serines 61 and 75 and threonine 87) in the N-terminal region and serine 265 in the ligand binding domain of mouse RXRα. However, JNK-mediated hyperphosphorylation of RXRα did not affect the transactivation properties of transfected RXRα homodimers or RXRα:RARα heterodimers in cultured cells [16]. In contrast, we and others have demonstrated that phosphorylation of RXRα by JNK signaling pathways is associated with reduced RXRα-dependent promoter activity [8,17]. Clearly, the consequences of RXRα phosphorylation are complex and poorly understood.

NR ligands and extracellular signal-mediated pathways can alter subcellular NR localization, some of which involves phosphorylation-dependent mechanisms [12,13,18-20]. The xenobiotic receptors, CAR (NR1I3) [19] and PXR (NR1I2) [20] are localized in the cytoplasm of mouse hepatocytes and translocated into the nucleus after administration of their respective ligands. GR and VDR are well-known to undergo ligand-dependent nuclear import in transfected cells [12,13,18,21]. In contrast to the well-described events leading to NR nuclear import, little is known about NR nuclear export, including RXRα. Perhaps the best understood example of cell signaling targeting of NR nuclear export is JNK-mediated phosphorylation of GR, as a means of terminating GR-mediated transcription [18]. Such a mechanism for RXRα has never been shown, although a reduction in nuclear RXRα protein levels has been demonstrated in an animal model of obstructive cholestasis induced by bile duct ligation, raising the possibility of nuclear export [22].

In these studies, we sought to determine whether alterations in RXRα-dependent hepatic gene expression seen in inflammation may be related to nucleo-cytoplasmic re-distribution of RXRα. LPS treatment resulted in the activation of hepatic JNK coinciding with marked reduction in nuclear RXRα levels, and the rapid appearance of RXRα in the cytosol. RNA levels of *RXRα *and six of its heterodimeric partners highly expressed in liver were analyzed after LPS treatment: *RXRα*, *RARα*, *FXR *(farnesoid X receptor) and *PPARα *(peroxisome proliferator-activated receptor) RNA levels were stable, whereas *CAR *(constitutive androstane receptor) and *PXR *(pregnane X receptor) RNA levels were markedly suppressed and *LXR *(liver X receptor) RNA was elevated. Hepatic RNA levels of multiple RXRα target genes whose expression depends upon adequate nuclear levels of RXRα were significantly reduced by LPS. This is likely to be a consequence of reduced nuclear binding activity of RXRα heterodimer pairs. Notably, the reduction in RXRα nuclear protein levels (~50%) quantitatively correlated with the reduction in RNA levels of RXRα target genes. Taken together, these studies indicate that post-translational modification and cellular re-distribution of RXRα coinciding with induction of cell signaling is a novel, broad-ranging, and rapid mechanism contributing to the negative hepatic APR phenotype in the inflamed liver.

## Results

### LPS activates hepatic JNK

**Figure 1:**
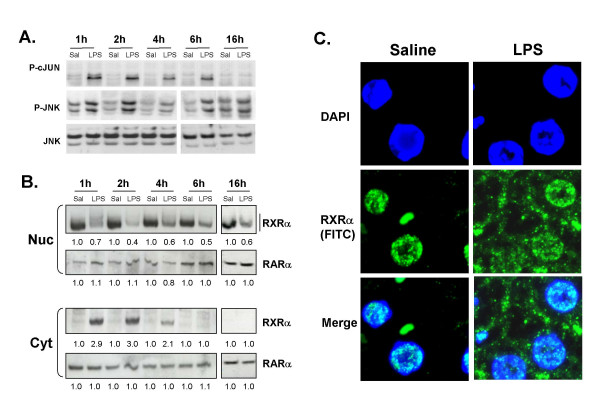
**LPS activates JNK and leads to rapid nuclear export of RXRα. **C57BL/6 male mice were injected IP with 0.9% saline (Sal) or 2 μg/g bw of *Salmonella *LPS. Livers were isolated at the indicated time-points and whole cell extracts were prepared. **A**. Phosphorylation of c-JUN (P-cJUN) and JNK (P-JNK) was determined by immunoblotting cell lysates with phospho-c-JUN and phospho-JNK antibodies respectively. Total JNK levels in the liver tissue (*JNK*) are shown in the *lower panels*. This data is representative of three animals per treatment group. **B. **Nuclear (Nuc) and cytosolic (Cyt) extracts were analyzed by immunoblotting with antibodies to RXRα and RARα to determine subcellular localization of RXRα. The extracts from 4 animals were combined to account for inter-animal variability. Note the high molecular weight smear in LPS-treated extractions (most evident at 1 h). Data quantified and normalized to saline-injected samples (set at 1.0). **C. **Immunofluorescent analysis of formalin-fixed mouse liver tissues after 1 h of saline or LPS treatment. The blue color indicates DAPI staining of the nuclei, the green color indicates RXRα detected with FITC-labeled secondary antibody, DAPI/FITC are the merged images. The saline and LPS-treated samples are represented in the left and right panels, respectively.

In order to determine the in vivo role for LPS-induced activation of hepatic JNK, we first established a time course for JNK activation, by measuring phospho-JNK and phospho-c-Jun levels. Liver whole cell extracts were prepared at various time points from 1–16 hours after injection with either LPS (2 μg/g bw) or vehicle (0.9% saline) (Fig. 1A). Phosphorylated JNK levels were maximal at 1–2 hours, and significantly higher than saline-injected animals at all time points studied. Total JNK levels did not vary between vehicle and LPS-treated samples. c-Jun is a direct substrate for phospho-JNK, and phospho-c-Jun levels are a well-described indicator of JNK activity [23]. LPS treatment led to maximal c-Jun phosphorylation at 1 to 2 hours, with a slight reduction at 4 and 6 hours, and was undetectable by 16 hours. Thus, in an animal model, LPS administration activates JNK signaling in the liver as early as 1 hour, with evidence for prolonged JNK activity lasting at least 6 hours.

### LPS treatment leads to the rapid reduction of nuclear RXRα protein levels concomitant with the appearance of RXRα in the cytoplasm

Treatment of HepG2 cells or primary rat hepatocytes with IL-1β leads to JNK-dependent repression of RXRα :RARα nuclear binding activity, with the consequent down-regulation of target gene expression [8]. However, whether such changes are observed in RXRα activity after LPS challenge in an animal model is unknown. As early as one hour after LPS treatment, the maximal point of JNK activation, nuclear RXRα levels were significantly reduced compared to control, and remained so for at least 16 hours after LPS treatment (Fig. 1B). RXRα was not present in the cytoplasmic fraction at any time point after saline treatment, yet was robustly evident within one to two hours after LPS treatment, and decreased thereafter. Interestingly, immunoblot analysis of nuclear RXRα revealed a slower migrating species after LPS treatment (most evident in the 1 hour LPS sample), suggestive of LPS-induced post-translational modification. Notably, hepatic RARα levels in both nuclear and cytoplasmic compartments were unchanged by LPS.

To confirm the intracellular localization of RXRα in liver, immunofluorescence staining was carried out in formalin-fixed liver tissues prepared from mice 1 h after saline or LPS injection (Fig. 1C). In LPS-treated mouse livers, RXRα was clearly observed in both the nucleus and cytoplasm of hepatocytes, whereas it remained exclusively nuclear in saline-treated controls. Thus, there is a rapid, dramatic, and specific subcellular re-distribution of hepatic RXRα in response to LPS-administration, coinciding with induction of cell signaling.

### Effect of LPS on steady-state mRNA levels of RXRα and its partners in the liver

**Figure 2:**
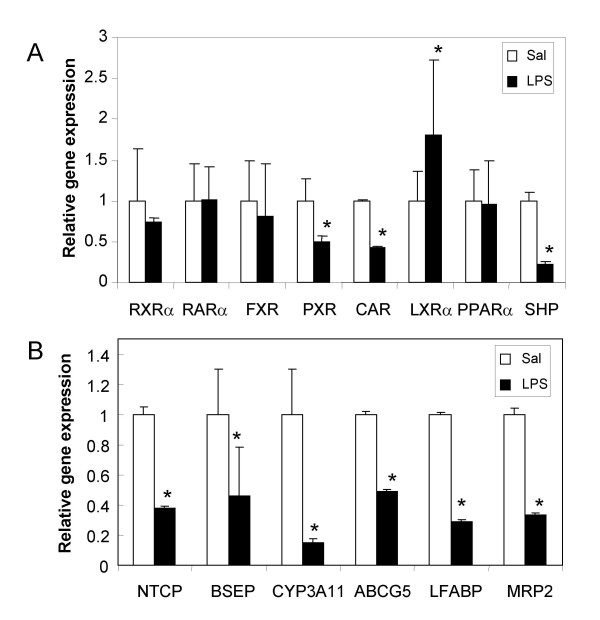
**Effects of LPS on RNA levels of NRs and RXRα target genes. **C57BL/6 mice were injected with 0.9% saline (*white bars*) or 2 μg/g bw of *Salmonella *LPS (*black bars*) for 16 hours (*n *= 6 per group). RNA was prepared from the livers and analyzed for **A. **NRs and **B. **RXRα target genes by real-time PCR. All data were presented as mean ± SD and standardized for GAPDH RNA levels. Expression in the saline-treated control animals was set to 1. The *asterisk*s indicate significant difference (p < 0.05). See supplemental information for primers and probes.

One possible explanation for reduced nuclear RXRα levels could be suppression of hepatic *RXRα *RNA expression, as seen in response to higher doses of LPS [24]. We investigated whether low dose LPS administration had an effect on the RNA levels of *RXRα*, six of its heterodimeric partners and SHP–all known to be involved in hepatic gene expression [25] (Fig. 2A). At 16 h after LPS administration, RNA levels for *RXRα, RAR*, *FXR *and *PPARα *were unchanged, whereas *PXR *and *CAR *RNA levels were reduced and *LXRα *RNA levels were increased (Fig. 2A). LPS-mediated down-regulation of *PXR *and *CAR *RNA levels in mice have been reported by Beigneux *et al*[26], however our results do not support the reduction in RNA levels of *RXRα*, *FXR *and *LXR *and *PPARα *seen by others, perhaps due to differences in the experimental model, LPS dose or mouse strain [24,27].

Hepatocyte-selective RXRα-null mice have impaired metabolic function, with reductions in CAR, FXR, LXRα, PPARα, and PXR target gene expression [28]. As examples of genes regulated by RXRα and its partners, we studied RNA expression of six sentinel hepatic genes regulated by various RXRα heterodimer pairs: *Ntcp *(RARα), *Bsep *(FXR), *Mrp2 *(CAR, FXR, PXR), *Cyp3A11 *(CAR, PXR), *Abcg5 *(LXRα) and *Lfabp *(PPARα) (Fig. 2B). RNA levels of all of these hepatic RXRα-regulated genes were significantly reduced by LPS treatment. *Ntcp*, *Bsep *and *Abcg5 *RNA levels decreased by 50–60%, *Cyp3A11 *RNA by 80%, while *Lfabp *and *Mrp2 *expression were each reduced approximately 60–70% after LPS treatment. The comparatively greater reduction in Cyp3A11 gene expression can be attributed to the combined effects of diminished PXR and CAR RNA expression along with reduced nuclear RXRα protein levels; both PXR & CAR activate Cyp3A11 gene expression (reviewed in [29]).

The orphan nuclear receptor SHP (small heterodimer protein, NR0B2) is known to repress the activities of RXRα and other NRs [11,29]. One possibility is that LPS-mediated suppression of hepatic genes could be mediated by the activation of the repressor, SHP [9]. However, this is unlikely, since LPS treatment dramatically reduced SHP RNA levels (Fig. 2A). Taken together, these studies indicate that the effect of LPS on hepatic RXRα-dependent gene expression is not due to reduced RXRα RNA levels or increased SHP–rather it appears to be a consequence of post-translational modification and rapid LPS-induced subcellular re-distribution of RXRα protein. This is in agreement that SHP-1 is a FXR/RAR target gene [11].

### Effect of LPS on DNA binding activity of Type II nuclear receptor pairs in liver

**Figure 3:**
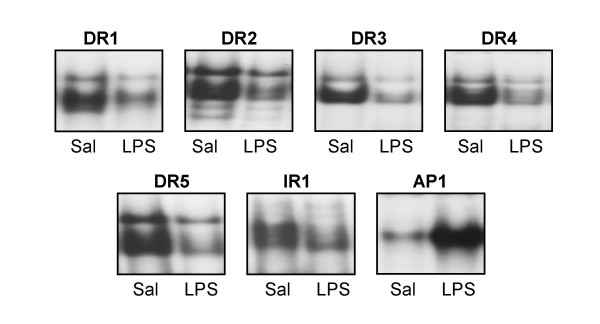
**LPS reduces binding activities of RXRα-containing heterodimer pairs to canonical DNA elements. **Electrophoretic mobility shift assay analysis of hepatic nuclear extracts prepared from C57BL/6 mice injected with control saline or 2 μg/g bw LPS for 16 h. Radiolabeled double-stranded DR1, DR2, DR3, DR4 and DR5 elements or a consensus AP1 element were employed (see Materials and Methods). The samples were electrophoresed through a 6% non-denaturing polyacrylamide gel, dried and analyzed by autoradiography.

In order to determine if reduced nuclear RXRα protein levels leads to impaired DNA binding activity of RXRα and its partners, electrophoretic mobility shift analyses were performed. Nuclear extracts were prepared from livers of saline or LPS-treated mice and incubated with oligonucleotides containing canonical DNA elements scanning Type II NR binding sites–direct repeats of the hexad AGGTCA, separated by 1 to 5 nucleotides (DR1-5), or an inverted repeat separated by 1 nucleotide (IR1) [30,31]. As Type II NRs, RXRα partners with either RARα, PPARα, PXR, CAR, LXR or FXR to bind to one or more of these sites (reviewed in [25]). At 16 h after LPS treatment, binding to all 6 RXRα-containing canonical sequences was significantly reduced in hepatic nuclear extracts from LPS-treated animals (~45–70% reduction) (Fig. 3), consistent with a diminished nuclear RXRα. Since the expression of PXR and CAR was reduced upon LPS administration (Fig. 2A), there was a more dramatic decrease in binding to their recognition elements, DR3 and DR4 (~70% reduction). Binding to the consensus AP1 DNA sequence was increased (~70%) upon administration of LPS; this serves as a positive control for JNK-mediated activation of hepatic inflammation as well as an indication of specificity of suppression of RXRα-heterodimer pair DNA binding (Fig. 3).

## Discussion

The negative hepatic APR is characterized by suppression of hepatic gene expression in response to inflammation and is well-modeled by LPS administration [32,33]. We hypothesized that reduced nuclear levels of RXRα after LPS administration would be manifested by broad alterations in RXRα-dependent gene expression across diverse physiological processes [28]. Our results demonstrate that LPS signaling induces rapid and profound reduction of hepatic nuclear RXRα protein levels, concomitant with appearance of RXRα in the cytoplasm, leading to subsequent reduction in the expression of RXRα-dependent hepatic genes.

Recent studies have led to a broader understanding of the molecular basis for the role of LPS in intracellular signaling and hepatic function [24,26,34]. Activation of monocytes/macrophages by LPS leads to the secretion of a number of proinflammatory cytokines such as TNFα, IL-1β, and IL-6 [2]. LPS-induced activation of Kupffer cells, the resident hepatic macrophages, triggers several crucial intracellular signaling pathways in hepatocytes, including stress-activated mitogen-activated protein kinases, extracellular signal-regulated kinase (ERK), JNK and p38 mitogen-activated protein kinase (p38 MAPK) [35]. Stress-activated protein kinases, mitogen-activated protein kinase kinase-4 (MKK4/SEK1) and its downstream mediator JNK was shown to directly phosphorylate RXRα[8,17]. Previous studies by our group[8] demonstrated that inhibition of the JNK signaling pathway completely blocked IL-1β-mediated suppression of RXRα-dependent *Ntcp *gene expression, thus implicating JNK to be a central player in inflammation-induced cholestasis. Most evident in the 1 hour sample are high molecular weight forms of RXRα, consistent with covalent post-translational modification (Fig. 1B), although the actual nature of this high molecular weight species is currently unknown and under investigation. One plausible interpretation of these data is that LPS-induced activation of JNK leading to phosphorylation and likely further modification of RXRα, triggering its transport from nucleus to cytoplasm, where it may be targeted for degradation. Phosphorylation has been shown to be involved in the degradation of RXRα :RARα heterodimers by proteasomes, thus providing a mechanism for JNK-mediated inhibition of RXRα-dependent target gene transactivation[14,15] RNA levels of RXRα were not affected by LPS, further supporting nuclear export of RXRα as a primary mechanism of suppression of hepatic genes during negative hepatic APR.

The interrelationship and roles played by JNK and phospho-RXRα are neither readily nor definitively explored in an in vivo model, especially using such a broadly-acting inflammatory agent like LPS. Hepatocytes and liver-derived HepG2 cells in culture respond to LPS-induced cytokines like TNFα and IL-1β by suppressing the expression of negative hepatic APR genes [8,24,26,36]. Recent work in our laboratory indicates that treatment of HepG2 cells with IL-1β leads to RXRα nuclear export, dependent upon JNK-mediated phosphorylation of select residues in RXRα (Zimmerman *et al*., manuscript in preparation). In transfected cells, nerve growth factor (NGF)-induced phosphorylation of the orphan nuclear receptor NGFI-B (Nur77) resulted in the translocation of RXR-NGFI-B complex out of the nucleus, indicating that distribution of RXR in these cells was regulated by NGFI-B [37]. The data presented here are the first to indicate that inflammation-mediated cell signaling leads to rapid subcellular redistribution of native RXRα, changing the previous impression of RXRα as a stable nuclear resident [38]. Finally, these findings indicate significant cross-talk between JNK-signaling and NR-mediated gene expression.

## Conclusions

Overall, we conclude that RXRα is rapidly exported out of the nucleus in response to LPS. RXRα, as an obligate heterodimer with other class II NRs, regulates the expression of a broad array of genes involved in critical metabolic pathways in the liver, many of which are impaired during the negative hepatic APR. This helps explain how inflammation-induced signaling can lead to rapid, diverse and multiple alterations in hepatic gene expression, which has implications for future therapeutic targets of both acute and chronic liver diseases.

## Materials and methods

### Materials

LPS (*Salmonella typhimurium*) was purchased from Sigma Chemical Co. (St. Louis, MO) and freshly diluted to the desired concentration in pyrogen-free 0.9% saline before injection. Anti-JNK (#9252), phospho-JNK (#9251) and phospho-cJUN antibodies (Ser 63) (#9261) (Cell Signaling, Beverly, MA); anti-RXRα (D-20) (#sc-553) and anti-RARα antibodies (#sc-551) (Santa Cruz Biotechnology, Santa Cruz, CA) were used according to manufacturer's instructions. [γ-^32^P]ATP was obtained from PerkinElmer Life Sciences (Boston, MA). Oligonucleotides were obtained from Sigma Genosys and Synthegen, Houston, TX. All reagents for real-time PCR were purchased from Applied Biosystems (Foster City, CA).

### Animals

Adult male (8–10 weeks) C57BL/6 mice (20–25 g) were purchased from Charles River Laboratories, (Wilmington, MA). The animals were maintained in a temperature- and humidity-controlled environment and were provided with water and rodent chow ad lib. Mice were given intraperitoneal injection with 2 μg/g body wt LPS (*Salmonella typhimurium*; Sigma Chemical Co., St. Louis, MO) in saline or saline alone. LPS in this dose range has been shown previously to induce cholestasis, maximally inhibit bile acid uptake, and significantly reduce *Ntcp *mRNA from 12 to 16 hours after injection, while not inducing hepatic damage [6,39]. Livers were removed at the time indicated in the figure legends (1 to 16 hours) after treatment. All animal protocols were approved by the Baylor College of Medicine Institutional Animal Care and Use Committee. Experiments were performed in triplicate and repeated three to four times.

### Preparation and analysis of nuclear and cytoplasmic and whole cell extracts

Nuclear and cytoplasmic extracts were prepared according to Itoh *et al*[18] Whole cell extracts were prepared according to Li *et al *[8]. Protein concentration was determined by BCA assay according to the manufacturer's protocol (Pierce, Rockford, IL). These fractions were analyzed by immunoblotting. Signals were developed by a standard enhanced chemiluminescence method following the manufacturer's protocol (Perkin Elmer Life Sciences, Boston, MA) and quantified by a densitometer using ImageQuant software.

### Immunofluorescent analysis

Livers were isolated from saline and LPS injected mice after 1 hour of treatment, fixed in 10% buffered neutral formalin overnight at 4°C and then stored in 70% ethanol. Fluorescent detection was performed by using anti-RXRα (D-20) antibody and fluorescein isothiocyanate (FITC)-labeled secondary antibody and nuclei was stained with 4'-6-diamidino-2-phenylindole (DAPI). Visualization was performed with a Deltavision Spectris Deconvolution Microscope System (Applied Precision, Inc.).

### Electrophoretic gel mobility shift assays

Nuclear extracts were prepared according to Timchenko *et al.*[40] with some modifications. Double-stranded oligonucleotide probes were end-labeled and purified according to standard procedures [41]. 10 μg of nuclear extracts were incubated on ice for 30 min with ^32^P end-labeled oligonucleotide as described previously [41]. The oligonucleotide sequences are provided in Table 1. After binding, the samples were electrophoresed through a non-denaturing 6% polyacrylamide gel, dried and exposed to x-ray film. In addition, gels were exposed to a PhosphorImager screen and quantified using a PhosphorImager and ImageQuant software.

### Real time quantitative PCR analysis

Total RNA was isolated from mouse liver tissues using the RNaesy kit from Qiagen. cDNA was synthesized from 7.5 μg of total RNA using the ProSTAR™ First-Strand RT-PCR Kit (Stratagene, La Jolla, CA). Real time quantitative PCR (RTQ-PCR) was performed using an ABI PRISM 7700 Sequence Detection System instrument and software (Applied Biosystems, Inc., Foster City, CA). Briefly, each amplification reaction (50 μl) contained 40–200 ng of cDNA, 300 nM of forward primer, 300 nM of reverse primer, 200 nM of fluorogenic probe and 25 μl of TaqMan^® ^Universal PCR master mix. PCR thermocycling parameters were 50°C for 2 min, 95°C for 10 min and 40 cycles of 95°C for 15 s, and 60°C for 1 min. Quantitative expression values were extrapolated from standard curves and were normalized to GAPDH. The sequences of the primers and probes were obtained from the literature [42] or purchased from Applied Biosystems, and are listed in Table 2.

## Abbreviations

The abbreviations used are: RXR, retinoid X receptor; RAR, retinoic acid receptor; FXR, farnesoid X receptor; PPAR, peroxisome proliferator-activated receptor; PXR, pregnane X receptor; CAR, constitutive androstane receptor; LXR, liver X receptor; SHP, small heterodimer partner; NR, nuclear receptor; GR, glucocorticoid receptor; PR, progesterone receptor; VDR, vitamin D receptor; JNK, c-Jun N-terminal kinase; AP-1, activator protein-1; Ntcp, sodium/taurocholate cotransporting polypeptide; Bsep, Bile salt export pump; Mrp2, multidrug resistance associated protein 2; Lfabp, liver fatty acid binding protein; Cyp3A11, cytochrome P450 3A11; PCR, polymerase chain reaction; GAPDH, glyceraldehyde-3-phosphate dehydrogenase; APR, acute phase response; DR, Direct Repeat; IR, Inverted Repeat; FITC, fluorescein isothiocyanate; DAPI, 4'-6-diamidino-2-phenylindole.
